# Identifying Patient Door-to-Room Goals to Minimize Left-Without-Being-Seen Rates

**DOI:** 10.5811/westjem.2015.7.25878

**Published:** 2015-10-20

**Authors:** Shea Pielsticker, Lori Whelan, Annette O. Arthur, Stephen Thomas

**Affiliations:** * University of Oklahoma College of Medicine, Oklahoma City, Oklahoma; † University of Oklahoma College of Medicine, Department of Emergency Medicine, Oklahoma City, Oklahoma; ‡ Hamad General Hospital, Department of Emergency Medicine, Doha, Qatar

## Abstract

**Introduction:**

Emergency department (ED) patients in the leave-without-being-seen (LWBS) group risk problems of inefficiency, medical risk, and financial loss. The goal at our hospital is to limit LWBS to <1%. This study’s goal was to assess the influence on LWBS associated with prolonging intervals between patient presentation and placement in an exam room (DoorRoom time). This study’s major aim was to identify DoorRoom cutoffs that maximize likelihood of meeting the LWBS goal (i.e. <1%).

**Methods:**

We conducted the study over one year (8/13–8/14) using operations data for an ED with annual census ~50,000. For each study day, the LWBS endpoint (i.e. was LWBS <1%: “yes or no”) and the mean DoorRoom time were recorded. We categorized DoorRoom means by intervals starting with ≤10min and ending at >60min. Multivariate logistic regression was used to assess for DoorRoom cutoffs predicting high LWBS, while adjusting for patient acuity (triage scores and admission %) and operations parameters. We used predictive marginal probability to assess utility of the regression-generated cutoffs. We defined statistical significance at p<0.05 and report odds ratio (OR) and 95% confidence intervals (CI).

**Results:**

Univariate results suggested a primary DoorRoom cutoff of 20′, to maintain a high likelihood (>85%) of meeting the LWBS goal. A secondary DoorRoom cutoff was indicated at 35′, to prevent a precipitous drop-off in likelihood of meeting the LWBS goal, from 61.1% at 35′ to 34.4% at 40′. Predictive marginal analysis using multivariate techniques to control for operational and patient-acuity factors confirmed the 20′ and 35′ cutoffs as significant (p<0.001). Days with DoorRoom between 21–35′ were 74% less likely to meet the LWBS goal than days with DoorRoom ≤20′ (OR 0.26, 95% CI [0.13–0.53]). Days with DoorRoom >35′ were a further 75% less likely to meet the LWBS goal than days with DoorRoom of 21–35′ (OR 0.25, 95% CI [0.15–0.41]).

**Conclusion:**

Operationally useful DoorRoom cutoffs can be identified, which allow for rational establishment of performance goals for the ED attempting to minimize LWBS.

## INTRODUCTION

Patients who leave the emergency department (ED) without being seen (LWBS cases) have been identified for many years as a high-risk group in terms of medical and operational outcomes (e.g*.* patient satisfaction). 1, 2 In the current era of ED crowding, there is growing concern about LWBS. 1, 3 The literature still identifies this area as being among the most important performance measures relating directly to the patient. 4–6 Progress is being reported for specific populations (e.g. psychiatric “holds,” pediatric patients), 7, 8 but broad-based efforts to eliminate LWBS have been summarized as having had success that is “modest, at best.” 9

One of the most intuitively obvious variables influencing LWBS rates is the time interval from the patient’s initial ED presentation to being seen by a physician. 10 Previous work focusing on ED length of stay (LOS) and related operations parameters have identified prolonged “wait times” as the most important factor driving LWBS rates. 11, 12

Using Emergency Severity Index (ESI) triage levels 13 to stratify patients, previous investigators have calculated desirable wait times to enable achievement of an LWBS goal of <2%. 11 ESI 3 (mid-range acuity on the 1–5 ESI scale) patients are recommended to have wait times of <45 minutes; ESI 4/5 patients’ wait time target should be <60 minutes. 11

We undertook the current study to characterize the relationship between DoorRoom and LWBS at one institution. The aim was to assess incremental DoorRoom timeframes, while adjusting for potential confounders, to determine optimal target DoorRoom for our ED.

## METHODS

There was no collection of patient identifiers, protected health information (PHI), or any clinical information on individual cases. The institution’s ethics review board exempted the study.

### Design

This was a retrospective analysis of data collected and entered into an administrative database, on a daily basis.

### Setting and time frame

The study was conducted over one year (8/2013–8/2014) at a 700-bed hospital with and annual ED census of 50,000. The ED is staffed by emergency medicine (EM)-boarded physicians and residents. LWBS cases are those patients who check in to the ED and who leave (with or without being triaged) before being seen by a physician. (The ED does not use mid-level providers.)

### Data collected and units of analysis

For this study, the unit of analysis was the “day.” The major variables of interest were daily LWBS and daily mean DoorRoom. For each of the 365 study days, we categorized LWBS dichotomously as to whether the institutional goal (<1%) was met. DoorRoom is the time elapsed between a patient’s being “signed in” to the ED to be seen, and that patient’s being placed in any ED room/bay to be seen by a physician. The ED information reporting system calculates DoorRoom mean times for each day; these daily means constituted this study’s DoorRoom variable. DoorRoom was collected as a continuous variable (i.e. a mean DoorRoom time was ascertained for each study day) and then analyzed as both a continuous and categorical variable as described below.

We incorporated daily ED census dichotomously, with the goal of adjusting for the study days on which there was low volume (and thus which historically have been associated with very low LWBS at the study ED). Using an a priori cutoff of 116 patients per day based upon historical data (this census number is roughly the bottom quartile of ED census spread in the study hospital), we coded the ED census covariate dichotomously to allow for the model to control for low-census days.

We also recorded daily inpatient hospital occupancy as a continuous variable. This occupancy rate was assessed and reported by the hospital’s information system at 0700 each morning. The study also included hospital operations data on daily means for ED LOS for all patients.

We assessed P=patient acuity using the ESI triage scale (1, most urgent, through 5 as least urgent). ESI categories 1 and 2 were categorized as “more urgent” acuity; the proportion of ESI 1 or 2 cases each day was used as a marker of overall ED acuity. As an additional representative of daily ED acuity level, we incorporated admission percentage for each study day into modeling.

### Analysis approach

For the main analysis, the dichotomous endpoint of interest (i.e. dependent variable) was “met LWBS goal” (i.e. coded as being met if the day’s LWBS was under 1%). The main independent variable was DoorRoom.

We initially assessed DoorRoom as a continuous variable. In order to assess the endpoint in operationally applicable categories each study day’s mean DoorRoom was also placed into one of a dozen ordinal “time bins.” The first time bin was delineated by DoorRoom times within 10 minutes. The second bin contained DoorRoom times of 11–15 minutes, the third bin DoorRoom times 16–20 minutes, and so on through the 12^th^ and final bin containing days with mean DoorRoom exceeding an hour.

We used skewness-kurtosis testing to assess data normality. For normally distributed data, central tendency is reported as mean±standard deviation (SD), with 95% confidence interval (CI) reported for the mean. For non-normal data, central tendency is reported as median with interquartile range (IQR).

Proportions data are reported with binomial exact 95% CIs. We assessed categorical data using chi-square testing or (if cell values fell below 5) Fisher’s exact test. The nonparametric trend test was used as an initial approach to assessment of whether there was a trend between increasing DoorRoom and LWBS.

After univariate testing, we used multivariate logistic regression to adjust for potential confounders while exploring the association between the major independent variable DoorRoom and the LWBS endpoint. Results were reported as odds ratio (OR) with 95% CI. Model comparisons and individual variables’ significance were performed using the likelihood ratio test.

To account for skewness in the continuous variables assessed, we calculated and used robust standard errors for 95% CI calculations around ORs. As model-building proceeded, potential confounders were reintroduced into the model for assessment as per standard approaches of assessing for >20% change in the β point estimate (regardless of statistical significance). 14

We assessed logistic regression model performance with the goodness-of-fit test of Hosmer and Lemeshow. 14 Classification performance was assessed by assessing the area under the curve (AUC) for the receiver operator characteristic (ROC) curve. We assessed the utility of previously identified DoorRoom cutoffs using the multivariate logistic regression model and predictive marginal probability analysis. 15

## RESULTS

### Descriptive statistics

Table 1 shows summary statistics for the study data. The median LWBS was just under the ED LWBS target maximum of 1% and the LWBS goal was met in 211 of 365 days (57.8%). Low-census days (ED census below 116) occurred 99 times, constituting 27.1% of the study n of 365 days. Other variables were not normally distributed and are reported in Table 1 with median and IQR.

### Basic analysis

Univariate analysis entailed separating the n=365 study days’ DoorRoom times into bins as previously described, and then for each time bin determining the proportion of the bin’s days for which the LWBS goal was met. For example, there were 48 days in which the mean daily DoorRoom fell between 16 and 20 minutes, and the LWBS goal was met in 41 (85.4%). Results are shown in Table 2.

The above analysis provides proportions of study days meeting the LWBS target for individual bins of time frames. There was a significant association between DoorRoom bin and likelihood of meeting the LWBS goal (p<0.001).

As seen in the Table 2 data, there is an initial fall-off in LWBS performance between groups 3 and 4. This suggests maximal benefit in setting DoorRoom target within 20 minutes (i.e. to prevent the fall-off associated with changing the target from 20 minutes to 25 minutes). After a continuing drop in LWBS performance as DoorRoom increases through 25, 30, and 35 minutes, there is another precipitous drop in LWBS performance as DoorRoom time moves from 35 to 40 minutes (between groups 6 and 7). This suggests that while the primary DoorRoom goal for the study facility should be 20 minutes, a secondary aim should be to keep DoorRoom within 35 minutes.

The next univariate analysis was intended to complement the individual time-bin analysis by providing information on the cumulative LWBS performance at incremental DoorRoom cutoffs. Whereas the Table 2 results depict LWBS performance by each time bin (e.g. group 3 corresponds to DoorRoom of 16–20 minutes), the cumulative analysis depicts summed LWBS performance for the time bins up to a given cutoff. For example, in Table 3, the third row corresponds to the cumulative LWBS performance for all time bins up to 20 minutes; the third row, therefore, also includes all study days with DoorRoom means within 20 minutes. The time groups in Table 3 are thus additive, with each cumulative group containing all of the time bins up to the group’s cutoff.

Table 3 confirms the utility of the primary DoorRoom cutoff at 20 minutes: the LWBS goal was met in 87.5% of study days with mean DoorRoom within 20 minutes. The cumulative-time in Table 3 suggests some utility of the secondary DoorRoom goal of ≤35 minutes, since at this cutoff the LWBS goal was met 77% of the time.

The final step in univariate analysis was to assess the association between DoorRoom and LWBS goals, to see if the positive association was in fact a trend (i.e. there was lower LWBS rate associated with decreasing DoorRoom). The nonparametric trend test revealed a statistically significant (p<0.001) trend between improving DoorRoom and LWBS, thus strengthening the case for proceeding with multivariate modeling.

### Analytic statistics: Logistic regression with endpoint “met LWBS goal (of <1%)”

After the univariate basic analysis revealed a clear relationship between improvement in DoorRoom and likelihood of meeting the institution LWBS goal, the next step was to build a logistic regression model that allowed further exploration of the DoorRoom/LWBS association while adjusting for covariates.

In the univariate logistic regression model, the DoorRoom group was significantly (p<0.001) associated with likelihood of meeting the LWBS goal. Moving up each group number (e.g. from Group 1 to Group 2) was associated with an 18% drop in odds of meeting the LWBS goal (OR 0.72, 95% [0.67–0.78]).

Bivariate logistic regression including the primary independent variable (DoorRoom group) and the other covariates was then executed with standard model-building cutoff of p<0.20 for inclusion in the model. 14 Adjustment for acuity (by ESI) and operations parameters of ED census and LOS resulted in exclusion (through non-significant p and through lack of confounding) of the covariates for day-of-week, admission percentage, and inpatient occupancy. Thus, the final model included the major independent covariate of interest (DoorRoom group), as well as covariates allowing adjustment for patient load (ED census) and acuity (proportion of ESI 1 or 2), as well as hospital and ED operations improvements over time (study month) and daily ED throughput (LOS) (Table 4).

With regard to the main predictor variable, the model indicates that each 5-minute increment in a day’s mean DoorRoom corresponds to a 23% reduction in the chances that the day’s LWBS will fall under the goal of 1%. The model’s AUC of 0.82 indicated “excellent” discrimination. 14 Goodness-of-fit testing failed to reject the null hypothesis of lack of fit (p=0.64).

**Figure 1:**
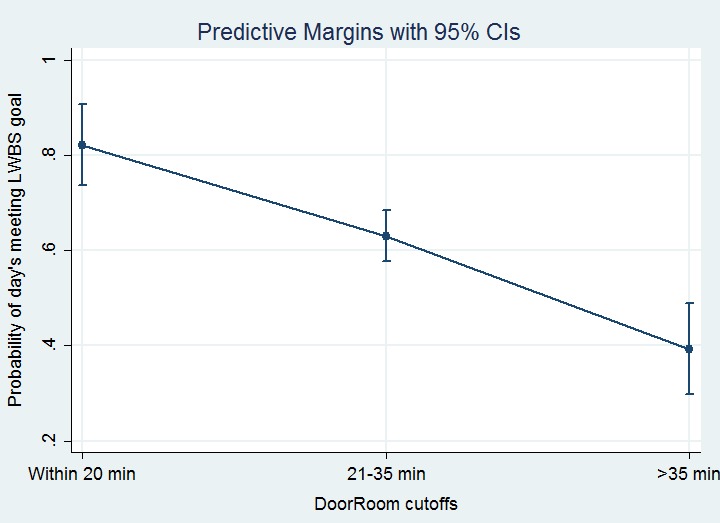
Multivariate logistic regression model predictions of likelihood, with 95% confidence intervals, of meeting left-without-being-seen (LWBS) goal (of <1%) at door-to-room (DoorRoom) cutoffs of 20 and 35 minutes. *CI*, confidence interval

Based upon the graphic suggestion of useful cutoffs at 20 and 35 minutes, we performed marginal analysis with the DoorRoom times categorized into categories of ≤20 minutes, 21–35 minutes, and ≥35 minutes. Increase of a day’s mean DoorRoom from within 20 minutes to the 21–35 minute time-frame was associated with a marked and statistically significant reduction in the chances of that day’s meeting LWBS targets; adjusting for other covariates in the final model, the OR for 21–35 time frame as compared to ≤20 minute time frame was 0.26 (95% CI [0.13–0.53], p<0.001). Similarly, prolonging a day’s DoorRoom mean from the 21–35 time frame to longer than 35 minutes was associated with another precipitous drop in likelihood of that day’s meeting the LWBS goal (OR adjusting for other covariates of 0.25 with 95% CI [0.15–0.41], p<0.001). Figure 1 depicts the probabilities of a given day’s meeting the institutional LWBS goal (<1%) at the cutoffs of mean DoorRoom within 20 minutes, 21–35 minutes, and >35 minutes. Lack of overlap of 95% CIs indicates these are reasonable cutoffs for operational planning.

## DISCUSSION

Medical outcomes problems (including medical-legal risk issues) are at the top of the list of LWBS concerns. 1, 16, 17 Other problems may include decreased patient satisfaction scores, 18 financial loss to the hospital, 3, 19 and even system-based efficiency issues such as repeated patient presentation after the initial LWBS episode. 20

A variety of factors can potentially impact LWBS. This study intended to adjust for a number of these factors, while focusing on one specific item: the time elapsed between the patient presentation to the ED and the patient being placed in a room (defined as DoorRoom for this study).

For various reasons we selected DoorRoom as the a priori endpoint of main interest for this study. First, it is intuitive. Second, although it’s clear that faster rooming of patients will decrease LWBS likelihood, the precise point that represents the best goal for DoorRoom is not known with certainty. Third, at the study institution, DoorRoom is a consistently measured and reported ED operations parameter. The more directly LWBS-relevant time interval of door-to-doctor is not accurately reported at the study institution.

Others have reported that LWBS can be significantly improved by placement of a physician or mid-level provider (MLP) at triage. 19, 21, 22 These programs seem to usually, although not invariably, result in a statistically significant reduction in LWBS. 23 Similarly favorable impact on LWBS has been reported with the institution of a “fast track” area of the ED, at which location less-critical patients are seen. 24 The study ED did not have a physician in triage, but did operate a fast-track daily from noon to midnight. Placement of a patient in a “room” was said to occur whether the room was a fast track bay or a room in the main ED. Regardless as to whether the initial evaluation occurs in triage (“out-front”), a fast track, or in the main ED, the major goal for those wishing to reduce LWBS seems to be minimizing the time interval between presentation and initial physician (or MLP) interaction. 17

Previous preliminary work suggested that goals for overall wait times should be set depending on ESI level; 45 minutes (for ESI 3 cases) or 60 minutes (for ESI 4 or 5 cases). 11 Studies around the world have demonstrated that triage acuity is regularly implicated as an important variable impacting LWBS rates. 5, 25–27 The internal and external validity of the current study is enhanced by the fact that much of the ED LWBS literature also uses the ESI to assess triage acuity. 1, 11

We undertook the current analysis to complement the existing literature, using a different multivariate methodology that adjusted for ESI as well as other operations parameters. Given the fact that previously suggested cutoffs for DoorRoom would result in high rates of failure of the study ED to meet LWBS goals, the current analysis was undertaken to try and identify DoorTime goals that would be operationally useful at the study institution. The primary aim was to identify an early cutoff, the meeting of which DoorRoom time would be associated with very high likelihood of meeting the LWBS goal. A secondary aim was determination as to whether there were an additional cutoff for a secondary DoorRoom time goal that would be associated with adequate (if not ideal) performance with regard to meeting LWBS goals.

The selection of time intervals and spacing, while executed a priori as part of study planning, was arbitrary. Operations group discussions prior to the study’s commencement identified 5-minute windows as the narrowest time frame for practical analysis. Experience at the study ED was that patients were so rarely “roomed” within five minutes that there would be no utility to establishing a “time bin” in the within-5-minute range. Therefore, the initial time bin was set at DoorRoom within 10 minutes. The next 11 categories were logically determined as succeeding 5-minute intervals were defined, but the last category (>60 minutes) was something of a catch-all. The reason this last DoorRoom time bin was set with such a large range was that the overall n of these longer-DoorRoom days was small and there was benefit in not having large numbers of sparsely populated time bins at the longer end of the DoorRoom spectrum. Furthermore, in study planning it was determined that there would be little to gain (in terms of setting ED operations goals) from proving the undesirability of taking over an hour to get patients roomed.

The study did not set out to identify what other parameters besides DoorRoom are related to LWBS. It is acknowledged that many variables influence LWBS, but incorporation of these covariates in the current study’s modeling was intended only to adjust for these factors and allow focus on the primary independent variable of interest: DoorRoom. The study’s concentration on DoorRoom was not intended to imply these other factors are not important, but rather to allow the establishment of data-driven goals for the study ED on a parameter – DoorRoom – that is clearly defined and easily discussed with staff. It is for this operational reason that the continuous variable DoorRoom was categorized into 5-minute windows for the study’s main analyses.

The covariate “study month” was statistically significant, and this finding warrants brief explanation. As is the case at many hospitals, multiple operations improvement measures were ongoing (or instituted) during the study period. Even measures that were not ED-based (e.g. increased surge capacity for bed availability) could still have downstream or indirect impact on ED operations and LWBS. Furthermore, ED operations improvement efforts continued throughout the study period on a number of fronts. As a coarse method of adjusting for these improvement efforts, the current analysis incorporated the chronological variable (“study month”), which was in fact statistically significant (showing that overall performance was improving solely as a function of ongoing work and passage of time).

There was a marked drop in the proportion of days meeting LWBS goals when DoorRoom exceeded 20 minutes, and a slightly lesser drop after 35 minutes. The univariate association between prolonging DoorRoom time and LWBS was confirmed in multivariate modeling, which also confirmed utility of the 20- and 35-minute DoorRoom cutoffs. The statistically significant cut points at both of these timeframes were also operationally significant: after each time cutoff the chances of meeting LWBS goals dropped by nearly 75%. The study methodology was insufficiently precise to support a claim that the 20- and 35-minute cutoffs are the only cutoffs that would be useful, but the results of the analysis do support institution of these cutoffs as a reasonable next step for the study institution’s ED operations group.

The numbers identified for the study ED remain to be assessed in a prospective analysis, and even if the cutoffs identified in this analysis work for the study ED, the utility of this report for other EDs lies more in its potential application of methodology, than in the particular results found at the study institution. In fact, some covariates in the study ED that were not identified as being statistically significant, have been specifically identified as important in other analyses. For example, weekend presentation has been identified as being independently associated with high LWBS in previous work (from locations as disparate as Australia and Switzerland). 28, 29 This finding was not replicated in the current analysis (p for day of week=0.53), emphasizing the importance of applying the analytic principles outlined here (and elsewhere) to one’s own patient population to determine the most important factors driving LWBS.

There is an additional issue with respect to extrapolation of this study’s results to other EDs. Because of the hospital and nursing administration focus on identifying a target to get patients “roomed,” DoorRoom was set as this study’s a priori endpoint. The small size of the ED during this project (30 beds plus an 8-bed fast track), meant that in our facility patients are seen within minutes of being “roomed.” In fact, the mean time interval from patients being placed in an ED room (main ED or fast track) and being seen by a physician was both rapid (11.1 minutes) and narrowly dispersed (95% CI for mean, 10.4–11.9). Therefore, the general results should be easily extrapolatable in other centers with similarly predictable association between DoorRoom and DoorDoctor.

## LIMITATIONS

The selection of variables assessed in the current analysis was somewhat limited, in that the operations database that was used as a data source included only limited information. Another major study limitation associated with the way the database is populated, is the use of the “day” as the unit of analysis. Another major study limitation was the use of the “day” as the unit of analysis for collecting LWBS and other information. It is certainly the case that, within a given day, there are variations in “risk” of high LWBS. The use of the day as unit of analysis was dictated by the data collection and reporting methods of the study ED’s administrative database, but it is acknowledged that follow-up studies should further narrow the analytic window and examine “within-day” LWBS.

Other study limitations are related to the study’s endpoint itself. First, the LWBS target endpoint of <1% in this study is arbitrary. Others have used different endpoints (e.g. <2%). 11 There is no concrete “correct” LWBS endpoint, but the <1% target set by the study hospital administration (well before this research project’s institution) is consistent with ED literature from hospitals of similar characteristics as the study facility. 30

An additional set of limitations regarding the study endpoint are related to the lack of any actual “impact” measurements in the current study. There was no information on actual financial or clinical impact of improving LWBS. As previously reported, the study hospital system uses an averaged-out “value” for an individual LWBS case (about $200), 3 but this average value is understood to be both imperfect and not necessarily generalizable. Therefore, the authors emphasize that the endpoint of “meeting LWBS goal” is the major aim of this analysis, with the extrapolation of the value of meeting that goal left for future discussion.

Other study limitations stress this report’s utility only as a preliminary report. First of all, the data analysis was based upon only a year of data at a single center. Since the results were statistically significant, the relatively low n of weeks was not viewed by the authors as a major constraint. However, there are plans to continually monitor and reanalyze these data as part of ongoing operational improvement efforts. Furthermore, the single-center nature of the study should give pause to those considering extrapolation of the results to different settings.

## CONCLUSION

Despite the limitations as noted, the study does provide some direction for forward-looking operations improvement efforts. First, the cutoffs identified are both consistent with common sense and also perceived to be reasonably achievable at the study institution. Using 20 minutes as a primary goal and 35 minutes as a secondary DoorRoom target, there are clearly delineated targets that can be easily communicated with staff during operations education. Follow-up analyses will determine the results of applying these operations goals at the study institution, and the study methods are offered as one potential route for other ED operations groups to analyze and optimize their performance with respect to the critical endpoint of minimizing LWBS.

## Figures and Tables

**Table 1:** Descriptive statistics for n=365 study days.

Variable	Median (IQR)
LWBS (%)	0.8 (0–1.7)
Admit (%)	25 (23–28)
Inpatient bed occupancy (%)	90 (85–94)
ESI Level 1 or 2 (%)	15.9 (13.0–18.9)
Time intervals (in minutes) from presentation (“door”) time	
Door to triage	16 (13–20)
Door to room	30 (20–44)
Door to departure from ED (i.e*.* LOS)	213 (190–238)

*LWBS*, left-without-being-seen; *ESI*, emergency severity index triage level (1 or 2 representing highest acuity)13; *ED*, emergency department; *LOS*, length of stay

**Table 2:** Likelihood of meeting the left-without-being-seen goal of <1%, by door-to-room (DoorRoom) time frame.

DoorRoom group (time frame)	Study days with mean DoorRoom in time frame	Study days in time frame for which LWBS goal was met; 95% confidence interval
1 (<10 minutes)	8	7/8 (87.5%, 47.3–99.7%)
2 (11–15 minutes)	40	36/40 (90.0%, 76.3–97.2%)
3 (16–20 minutes)	48	41/48 (85.4%, 72.2–93.9%)
4 (21–25 minutes)	52	37/52 (71.2%, 56.9–82.9%)
5 (26–30 minutes)	40	24/40 (60.0%, 43.3–75.1%)
6 (31–35 minutes)	36	22/36 (61.1%, 43.5–76.9%)
7 (36–40 minutes)	32	11/32 (34.4%, 18.6–53.2%)
8 (41–45 minutes)	26	9/26 (34.6%, 17.2–55.7%)
9 (46–50 minutes)	20	5/20 (25.0%, 8.7–49.1%)
10 (51–55 minutes)	18	7/18 (38.9%, 17.3–64.3%)
11 (56–60 minutes)	14	4/14 (28.6%, 8.4–58.1%)
12 (>60 minutes)	31	8/31 (25.8%, 11.9–44.6%)
All study days	365	211/365 (57.8%, 52.6–62.9%)

*LWBS*, left-without-being-seen

**Table 3:** Probability of meeting left-without-being-seen goal (<1%) at different door-to-room (DoorRoom) cutpoints.

DoorRoom cumulative time group	DoorRoom time	Study days with mean DoorRoom in time frame	% study days with mean DoorRoom within cumulative timeframe, that met LWBS goal (with 95% confidence interval)
UpTo10	≤10 minutes	8	7/8 (87.5%, 47.3–99.7%)
UpTo15	≤15 minutes	48	43/48 (89.6%; 77.3–96.5% )
UpTo20	≤20 minutes	96	84/96 (87.5%; 79.2–93.4%)
UpTo25	≤25 minutes	148	121/148 (81.8%; 74.6–87.6%)
UpTo30	≤30 minutes	188	145/188 (77.1%; 70.4–82.9%)
UpTo35	≤35 minutes	224	167/224 (74.6; 68.3–80.1%)
UpTo40	≤40 minutes	256	178/256 (69.5%; 63.5–75.1%)
UpTo45	≤45 minutes	282	187/282 (66.3%; 60.5–71.2%)
UpTo50	≤50 minutes	303	193/303 (63.7%; 58.0–69.1%)
UpTo55	≤55 minutes	320	199/320 (62.2%; 56.6–67.5%)
UpTo60	≤60 minutes	334	203/334 (60.8%; 55.3–66.0%)
AllTimes	All study days	365	211/365 (57.8%; 52.6–62.9%)

*LWBS*, left-without-being-seen

**Table 4:** Variables included in final logistic regression model: association of door-to-room (DoorRoom) time bin group with likelihood of meeting left-without-being-seen goal.

Variable	OR (95% CI)	p
DoorRoom group (incremental time bin)	0.77 (0.68–0.88)	<0.001
Study month	1.21 (1.13–1.31)	<0.001
Low ED census	2.2 (1.16–4.36)	0.015
% low-acuity (triage index 1 or 2)	1.08 (1.01–1.14)	0.017
ED length of stay	0.99 (0.97–1.00)	0.026

*ED*, emergency department; *OR*, odds ratio
